# A Novel Prognostic Signature Associated With the Tumor Microenvironment in Kidney Renal Clear Cell Carcinoma

**DOI:** 10.3389/fonc.2022.912155

**Published:** 2022-07-04

**Authors:** Dongchen Pei, Chaojie Xu, Dong Wang, Xiaoxue Shi, Yurui Zhang, Yi Liu, Jianhua Guo, Nan Liu, Haipeng Zhu

**Affiliations:** Department of Urology, the Fifth Affiliated Hospital of Zhengzhou University, Zhengzhou University, Zhengzhou, China

**Keywords:** kidney renal clear cell carcinoma, bioinformatics, tumor microenvironment, prognosis signature, NMF (nonnegative matrix factorization)

## Abstract

**Background:**

The tumor microenvironment (TME) is a complex and evolving environment, and the tumor immune microenvironment in kidney renal clear cell carcinoma (KIRC) has a strong suppressive profile. This study investigates the potential prognostic role and value of genes of the tumor microenvironment in KIRC.

**Methods:**

The transcriptome sequencing data of 530 cases and 39 cases of KIRC and the corresponding clinical prognosis information were downloaded from TCGA data and GEO data, respectively, and TME-related gene expression profiles were extracted. A prognostic signature was constructed and evaluated using univariate Cox regression analysis and LASSO regression analysis. Gene set enrichment analysis (GSEA) was used to obtain the biological process of gene enrichment in patients with high and low-risk groups.

**Results:**

A prognostic signature consisting of eight TME-related genes (LRFN1, CSF1, UCN, TUBB2B, SERPINF1, ADAM8, ABCB4, CCL22) was constructed. Kaplan-Meier survival analysis yielded significantly lower survival times for patients in the high-risk group than in the low-risk group, and the AUC values for the ROC curves of this prognostic signature were essentially greater than 0.7, and univariate and multifactorial Cox regression analyses indicated that the risk score was independent risk factors for KIRC prognosis. GSEA analysis showed that immune-related biological processes were enriched in the high-risk group and that risk values were strongly associated with multiple immune cell scores and immune checkpoint-related genes (PDCD1, CTLA4).

**Conclusions:**

The prognostic signature can accurately predict the prognosis of KIRC patients, which may provide new ideas for future precision immunotherapy of KIRC.

## Introduction

Renal cell carcinoma (RCC) is a common genitourinary malignancy that causes nearly 170,000 deaths each year (1). Kidney renal clear cell carcinoma (KIRC) is the predominant histological type, accounting for approximately 75% of all RCC cases (2). The main treatment modality for KIRC is currently surgical resection, and early-stage patients can achieve good results with surgery. However, in patients with advanced KIRC with recurrence and metastases, surgery is difficult, recurrence rates are high, conventional radiotherapy is not sensitive, and the prognosis is usually very poor (3). However, no reliable biomarkers have been identified to predict the prognosis of KIRC patients (4).

The tumor microenvironment (TME) is a complex and evolving environment that varies in composition depending on the tumor type and consists mainly of stromal cells, immune cells, and the extracellular matrix (ECM), with immune cells being a key component of the TME (5). Renal cancer TME is also a dynamic system that plays a key role in driving immune escape (6). Unlike other tumor types, the tumor immune microenvironment of KIRC is characterized by a high degree of immune cell infiltration, with the highest degree of T-cell infiltration (7). Chevrier et al. also found that T cells and tumour-associated macrophages (TAMs) were the major immune cell populations in KIRC, accounting for an average of 51% and 31% respectively, with CD8+ T cells being mostly depleted and functionally deficient (8). A growing number of studies have shown that the therapeutic efficacy of immune checkpoint inhibition (ICIs) is closely related to the components of the TME. Recent clinical trial results have shown that ICIs combined with anti-angiogenic agents, or a combination of different ICIs are superior to monotherapy, making it the most effective treatment strategy for advanced KIRC today (9). However, immunosuppressive cell subsets and molecules in the TME can lead to the insensitivity of KIRC to immunotherapy. Therefore, it is essential to elucidate the occurrence and development of KIRC and TME-related genes, and transcriptome sequencing can be used to screen and identify potential targets for disease treatment (10).

It also has been shown that cancer cells and tumor-infiltrating immune cells in TME play an important role in regulating cancer progression. They play an important role in determining the type of malignancy. Tumor-infiltrating lymphocytes, including T cells and B cells, are an important class of cells in TME. CD4+ helper T cells and cytotoxic CD8+ T cells play an important role in tumor prevention by targeting antigenic tumor cells, and CD8+ T cells are associated with better clinical outcomes and immunotherapeutic responses in many cancers. In addition, it was recently observed that tumor-associated B cells play an important role in the immune system by producing antibodies and presenting antigens that can predict survival and response to immune checkpoint blockade therapy. In addition, an association between TME genetic signature and lower survival was observed in KIRC patients, and tumor-associated macrophage and T cell phenotypes were found to correlate with clinical outcomes. These observations highlight the importance of analyzing TME, including immune cell variability, to identify target tumors for each specific treatment and to design new effective cancer therapies.

Given the high heterogeneity of TME in KIRC (11), understanding TME-related gene expression changes has clinical implications for clarifying the prominent molecular characterization and prognosis of KIRC. Therefore, we identified two subtypes with different TME characteristics based on TME-related genes and constructed a prognosis-related predictive scoring signature to provide a molecular basis for the pathogenesis and treatment of KIRC.

## Materials and Methods

### Data Sources and Processing

Gene expression data and clinical information on KIRC were downloaded from The Cancer Genome Atlas (TCGA) database (72 normal samples and 539 tumor samples), and after excluding patients with no recorded survival time, 530 KIRC tumor samples were finally included, defined as an entire TCGA cohort. The dataset GSE29609 (39 tumor samples) containing the KIRC for survival time was also downloaded from the Gene Expression Omnibus (GEO) as a model test set to validate the predictive power of the signature. We eliminate the non-conforming data through quality control. Then all data expression values are transformed to a comparable level for subsequent analysis by standardization of the data. The main standardization method is quantile standardization. Based on previous studies (12–14), 4061 TME-related genes were obtained. TME-related genes were extracted, and differential genes were analyzed using the “limma” package in R. A threshold value of P < 0.05 and |log2FC| > 1 was defined to identify differentially expressed genes (DEGs).

### Identification of Molecular Subtypes of TME-Related DEGs

The differentially expressed TME-associated genes in KRIC tissues and adjacent tissues were screened, and the screened differential genes and their expression were organized into a gene expression matrix with a corrected p<0.05 and the absolute value of differential expression multiplicity >1 (FDR<0.05 and | log2Fold Change|>1) was set as the threshold value, and the “NMF” package (15) was used to extract the biological correlation coefficients of the data in the above-mentioned differential gene expression matrix. The samples were grouped by organizing the genes and samples to capture the internal structural features of the data. When clusters k = 2, the clusters showed appropriate performance and stability, resulting in two subtypes (C1 and C2). And survival prognosis analysis, including overall survival (OS) and progression-free survival (PFS), was performed using ‘survival’.

### Differences in Immunological Characteristics of Different Subtypes

A comparative analysis of the immune profile of patients with different subtypes was performed to clarify the differences in the immune profile of each subtype. Immune cells are an important component of the tumor immune microenvironment, and we used the “MCPcounter” R package (13) to calculate the immune scores of 10 immune cells, including CD8+ T cells, cytotoxic lymphocytes, fibroblasts, monocytic lineage, myeloid dendritic cells, NK cells, T cells, neutrophils, endothelial cells, B lineage. The immune scores of immune cells were compared between C1 and C2.

### Prognostic Signature Construction and Validation

Using the ‘caret’ package (16), the 530 TCGA samples were randomly divided into the most appropriate TCGA training cohort and TCGA testing cohort using a 3:7 subgroup sampling ratio, with both groups being similar in terms of clinical characteristics. The TCGA training cohort was used for signature construction, and the TCGA testing cohort and the entire TCGA cohort were used to internally validate the predictive power of the signature, while external validation was performed in the GSE29609. In the TCGA training cohort, the differential genes were subjected to univariate Cox regression, set at P ≤ 0.05, to screen for the corresponding TME-related prognostic genes. LASSO regression and cross-validation were performed using the “glmnet” package to obtain the optimal gene set, and 10-fold cross-validation was used to construct a prognostic risk score signature with the formula: risk score = risk gene expression_1_ × coef_1_ + risk gene expression_2_ × coef_2_ +… + risk geneexpression × coef_n_ (coef was the risk coefficient). The prognostic signature was used to calculate the risk values for each sample of TCGA training cohort, TCGA testing cohort, entire TCGA cohort, and GSE29609, respectively. The risk scores of the TCGA training cohort groups were ranked from lowest to highest and patients were divided into low- and high-risk groups based on the median. Kaplan-Meier analysis and log-rank tests were used to assess the survival of the two groups of patients, and receiver operating characteristic (ROC) curve analysis and decision curve analysis (DCA) were used to evaluate the predictive ability of the signature. We also established a nomogram to better predict the prognosis of KIRC patients, and the concordance index (C-index) and DCA were used to evaluate the performance of the nomogram. To demonstrate the superior performance of the signature developed in this study, we compared the signature with three recently published prediction models (17–19).

### Independence Analysis of Prognostic Signature and Clinical Characteristics

In addition, the predictive power of the predictive signature and other clinical characteristics (Age, gender, grade,stage, TNM staging) were compared using univariate Cox regression analysis and multivariate Cox regression analysis. In the entire TCGA cohort, Kaplan-Meier survival analysis stratified by TNM staging and stage staging was performed on KIRC patients to further validate the performance of the prognostic signature.

### Gene Set Enrichment Analysis for the Prognostic Signature and its Significance in Clinical Treatment

GSEA was used to analyze the enrichment of biological processes (BP) in the high-risk and low-risk groups, using GSEA 4.0.3 software for GSEA analysis. There is a strong relationship between the effectiveness of immunotherapy and the tumor immune microenvironment. Therefore, we calculated the correlation between the risk scores and the MCP algorithm for the immune score of immune cells using the Spearman method. We further explored the correlation of risk scores with the expression of representative genes of immune checkpoints, DNA replication, mismatch repair, and epithelial-mesenchymal transition (EMT).

## Results

### Correlation Between Immune Scores and KIRC Subtypes

**Figure 1:**
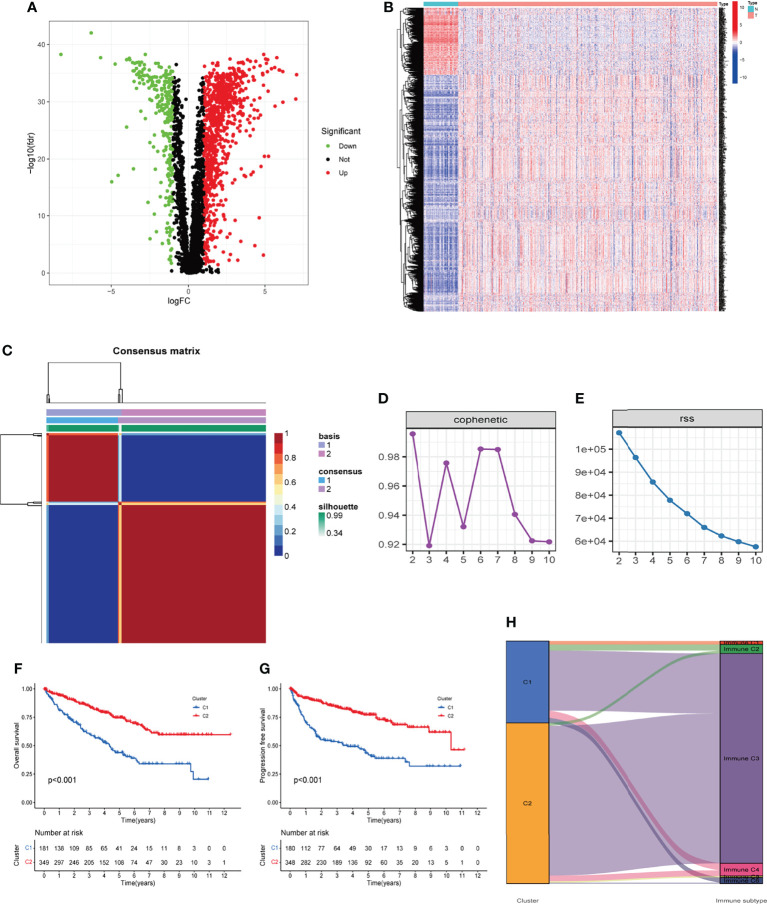
Two clusters were identified based on TME-related differentially expressed genes. **(A, B)** Volcano and heatmap of the distribution of differentially expressed genes, with P < 0.05 and |log2 FC| > 1.0 as cut-off values. **(C)** Consensus map of 530 cases of KIRC *via* the NMF algorithm. **(D, E)** Factorization rank for k = 2–10. **(F, G)** The Kaplan‐Meier survival curve showed the OS and PFS of the two subtypes of patients. **(H)** Percentage of the four immune subtypes accounting for each of the two clusters. TME, tumor microenvironment; KIRC, kidney renal clear cell carcinoma; NMF, non-negative matrix factorization; OS, overall survival; PFS, progression-free survival.

A total of 530 KIRC samples from TCGA and 39 samples from GSE29609 were included after pre-treatment (**Table 1**). The TME-related transcriptomic data downloaded from the TCGA database for KIRC were analyzed for differential expression in cancer versus para cancer groupings, and the results are shown in (**Figures 1A, B** and **Table S1**). In these TME related DEGs, the NMF algorithm is used to find that the clustering result is the best when k=2, so we build two clusters: Cluster1 and Cluster2 (**Figures 1C** and **S1**). Cophenet index is a measure of correlation between the distance of points in the feature space and the distance on the tree graph. Usually, it obtains all possible pairs of points in the data and calculates the Euclidean distance between these points. With this analysis, we determined that the C1 and C2 groupings are two independent clusters (**Figures 1D, E**). A comparison of OS and PFS between the two clusters showed significant differences (**Figures 1F, G**), with patients in the Cluster2 subtype having a longer survival time and a better prognosis. Our molecular typing results were compared with six immune subtypes of international translational immune typology of solid tumors. The results are shown in **Figure 1H**. To explore the relationship between the immune infiltration of KIRC and each subtype, the immune scores of individual immune cells were calculated for each sample by the MCP-counter algorithm and were compared.

**Table 1:** Clinicopathological characteristics of KIRC patients from the TCGA and GEO databases.

Characteristics	TCGA-KIRC cohort N = 537	GSE29609 N = 39
**Age**		
**<=65**	352(65.55%)	22 (56.41%)
**>65**	185(34.45%)	17 (43.58%)
**Gender**		
**Female**	191(35.56%)	˜
**Male**	346(64.43%)	˜
**Grade**		
**High**	286(53.25%)	26 (66.67%)
**Low**	244(45.43%)	13 (33.33%)
**Unknow**	8(0.01%)	0 (0.00%)
**Stage**		
**I-II**	326(60.70%)	74 (79.57%)
**III-IV**	209(38.91%)	11 (11.83%)
**Unknow**	3(0.01%)	8 (8.60%)
**T**		
**T0-T2**	344(64.05%)	16 (41.02%)
**T3-T4**	193(35.94%)	23 (58.97%)
**Unknow**		
**M**		
**M0**	426(79.32%)	26(66.67%)
**M1**	80(14.89%)	13(33.33%)
**Unknow**	32(0.06%)	0 (0.00%)
**N**		
**N0**	240(44.69%)	31(79.48%)
**N1**	17(0.03%)	5(12.82%)
**N2**	0 (0.00%)	3(0.08%)
**Unknow**	280(52.14%)	0 (0.00%)
**Survival status**		
**Alive**	~	22(56.41%)
**Dead**	~	17(43.58%)
**The median follow-up time** **(year)**	~	3.63

**Figure 2:**
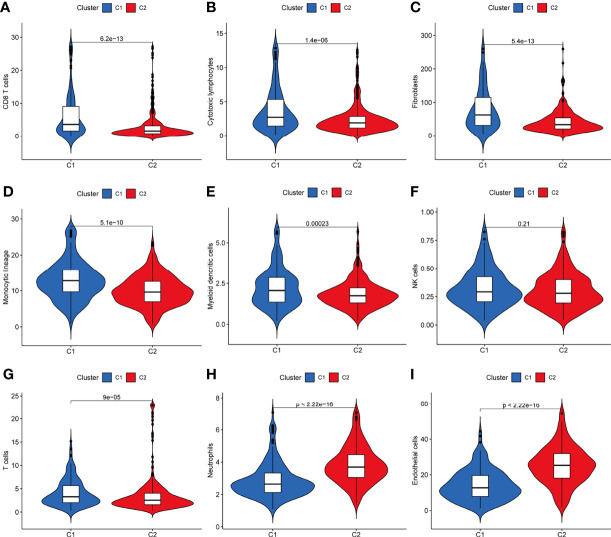
Differences in the distribution of immune cell content *via* the MCP-counter algorithm between the two clusters of KIRC. **(A–I)** Patients in the C1 group had higher immune scores of CD8+ T cells, cytotoxic lymphocytes, fibroblasts, monocytic lineage, myeloid dendritic cells, T cells, and lower immune scores for neutrophils and endothelial cells (P < 0.05). MCP-counter, microenvironment cell populations-counter; KIRC, kidney renal clear cell carcinoma.

The results showed that patients in the Cluster1 group had higher immune scores of CD8+ T cells, cytotoxic lymphocytes, fibroblasts, monocytic lineage, myeloid dendritic cells, T cells (P<0.05, **Figures 2A–G**), and lower immune scores for neutrophils and endothelial cells (P<0.05, **Figures 2H–I**).

### Establishment and Validation of the TME-Related Signature

**Figure 3:**
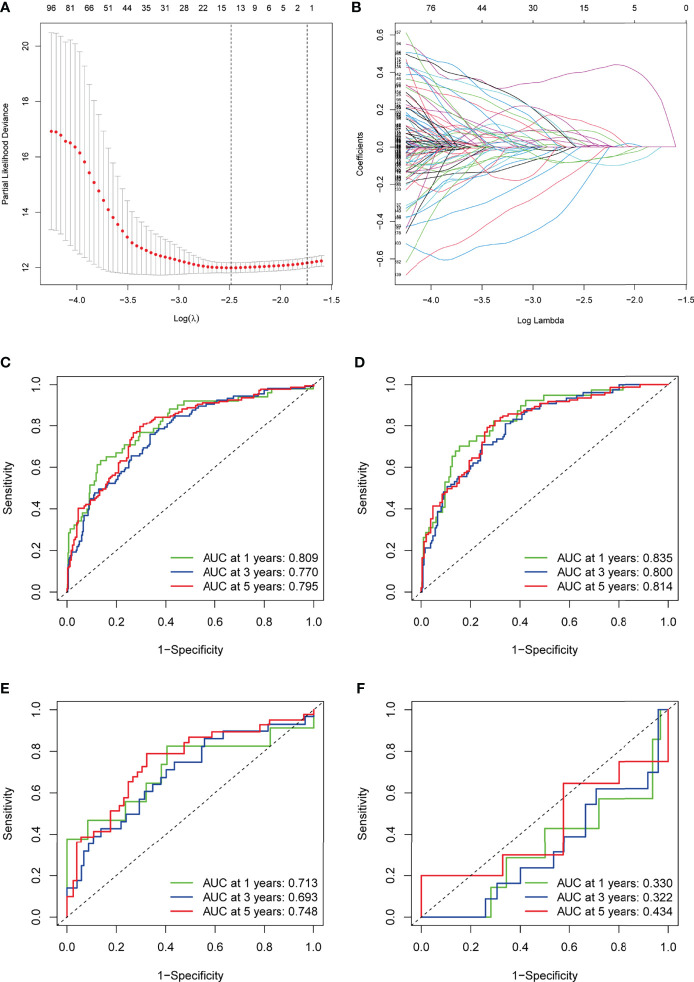
Construction of the TME-related signature for KIRC. **(A)** LASSO coefficients of the prognostic TME-related gene. **(B)** The LASSO model parameters (λ) were selected using a minimum criterion of ten cross-validations. **(C–F)** ROC curves for prognostic models of the entire TCGA cohort, TCGA training cohort, TCGA testing cohort, and GSE29609. TME, tumor microenvironment; KIRC, kidney renal clear cell carcinoma; LASSO, least absolute1 shrinkage and selection operator; ROC, receiver operating characteristic.

The entire TCGA cohort of 530 cases was randomly divided into the most appropriate training cohort (374 samples) and the test cohort (156 samples) in a 3:7 subgroup sampling ratio and the two groups were similar in terms of clinicopathological characteristics (Age, gender, grade, stage, TNM staging), as shown in **Table 2**. In the TCGA training cohort, the above DEGs were subjected to univariate Cox regression, and P ≤ 0.05 was set to obtain genes significantly associated with the prognosis of KIRC patients. A total of 8 genes were identified for the construction of the prognostic signature (**Figures 3A, B**) by removing over-fitted genes through LASSO regression analysis. These genes include LRFN1, CSF1, UCN, TUBB2B, SERPINF1, ADAM8, ABCB4, CCL22. The main biological functions involved include chemotaxis of some non-characterized immune cells as well as lymphocytes. Most tissue macrophages and osteoclasts are regulated by colony-stimulating factor-1 (CSF-1, also known as macrophage CSF). LRFN (leucine-rich repeat and fibronectin type-III domain-containing protein) recognizes bacteria and promotes hemocytic phagocytosis. CCL22 expression by dendritic cells (DCs) promotes the formation of cell–cell contacts and interaction with regulatory T cells (T reg) through their CCR4 receptor. These genes also include some of the genes that make up the basic cellular units, including Tubulin and the extracellular matrix. The TUBB2B gene provides instructions for making one version of a protein called beta-tubulin (β-tubulin). This protein is part of the tubulin family of proteins that form and organize cell structures called microtubules.

**Table 2:** Comparison of TCGA training and testing cohort.

Characteristics	TCGA Testing Cohort N = 107	TCGA Training Cohort N = 252	*p*-Value
**Age**			0.9888
**<=65**	103 (66.03%)	245 (65.51%)	
**>65**	53 (33.97%)	129 (34.49%)	
**Gender**			0.0805
**Female**	64 (41.03%)	122 (32.62%)	
**Male**	92 (58.97%)	252 (67.38%)	
**Grade**			0.8723
**High**	80 (51.28%)	201 (53.68%)	
**Low**	74 (47.44%)	167 (44.66%)	
**Unknow**	2 (1.28%)	6 (1.60%)	
**Stage**			0.3219
**I-II**	95 (60.90%)	227 (60.69%)	
**III-IV**	60 (38.46%)	145 (37.77%)	
**Unknow**	1 (0.64%)	2 (0.53%)	
**T**			0.8982
**T0-T2**	99 (63.46%)	241 (66.44%)	
**T3-T4**	57 (36.53%)	133 (35.56%)	
**Unknow**	0 (0.00%)	0 (0.00%)	
**M**			0.116
**M0**	132 (84.62%)	288 (77.01%)	
**M1**	17 (10.9%)	61 (16.31%)	
**Unknow**	7 (4.49%)	25 (6.68%)	
**N**			0.4317
**N0**	74 (47.44%)	165 (44.12%)	
**N1**	7 (4.49%)	9 (2.41%)	
**Unknow**	75 (48.08%)	200 (53.48%)	

**Figure 4:**
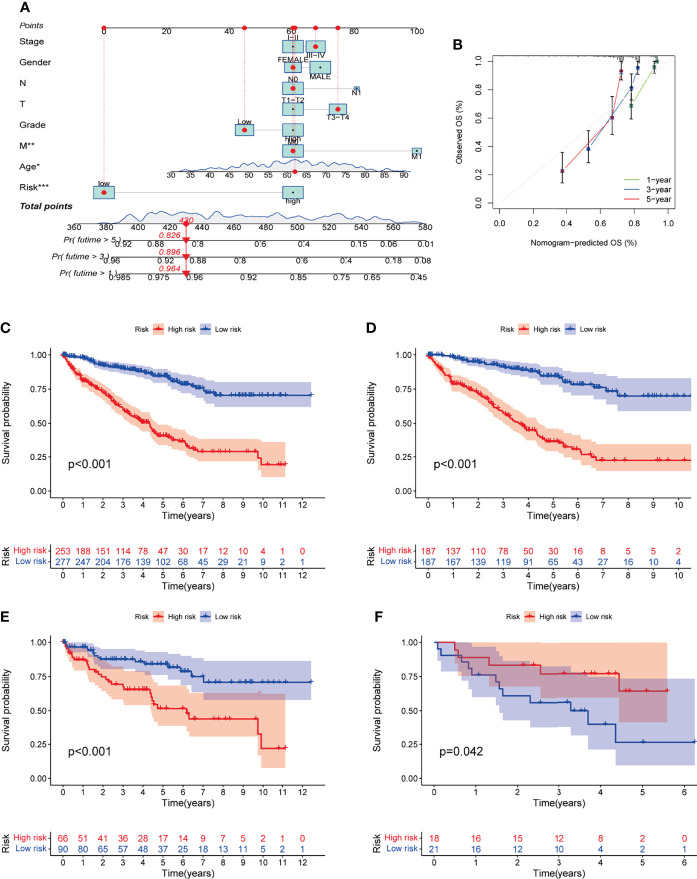
Establishment and evaluation of nomogram. **(A)** Nomogram was constructed by the prognostic model risk score, patient age, gender, stage, grade, and TNM Staging based on multivariate regression analysis. **(B)** 1-year, 3-year and 5-year calibration curves. **(C-F)** Kaplan-Meier survival curves for prognostic models of entire TCGA cohort, TCGA training cohort, TCGA testing cohort, and GSE29609.

Based on the expression of each gene and the corresponding coefficients, the following formula was constructed to calculate the risk score: Risk score = (LRFN1 * 0.433) + (CSF1 * 0.448) + (UCN * 0.376) + (TUBB2B * 0.190) + (SERPINF1 * 0.201) + (ADAM8 * 0.271) + (ABCB4 * -0.579) + (CCL22 * -1.055). To assess the accuracy of our signature predictions, we introduced a time-dependent ROC curve analysis, which showed that the prognostic signature showed low accuracy in GSE29609 (**Figure 3F**). However, the entire TCGA cohort, TCGA training cohort, and TCGA testing cohort showed higher accuracy, with AUC values almost all greater than 0.7 in predicting 1-, 3-, and 5-year survival (**Figures 3C–E**). Prognostically, as shown in **Figures 4C–E**, patients at higher risk had a worse prognosis compared with those in the lower risk group (P<0.001), and we obtained the same result in the GSE29609 dataset (**Figure 4F**). To provide clinicians with a quantitative method to predict the prognosis of patients with KIRC, we further created a nomogram (**Figure 4A**), and we assessed the predictive efficacy of the nomogram by calibration plot (**Figure 4B**). Based on the calibration plot, we can find that the 1-, 3-, and 5-year survival rates predicted by the nomogram overlap well with the actual survival rates of KIRC patients, which indicates that the nomogram can better predict the survival rates of KIRC patients.

### The TME-Related Signature Was an Independent Predictor of Prognosis

**Figure 5:**
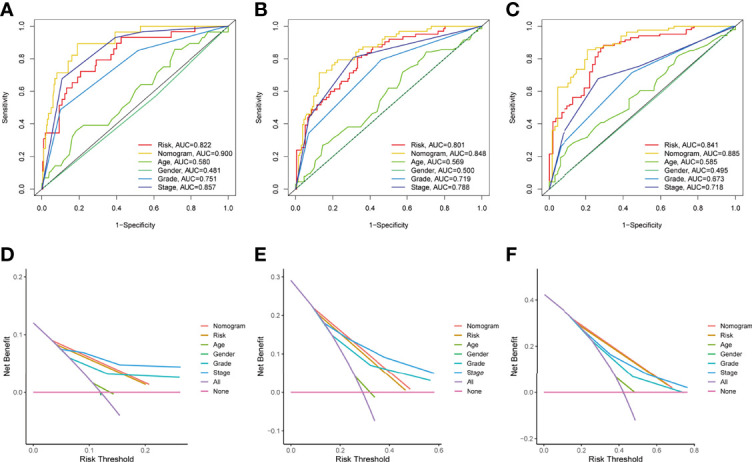
Independent predictive analysis of TME related characteristics. In the whole TCGA cohort, ROC curves of our characteristics and other clinical indicators were analyzed for 1 year, 3 years and 5 years respectively **(Figures A–C)**. DCA curve analysis of our characteristics and other clinical indicators, the time is also one year, three years and five years **(Figures D, E)**. Tumor microenvironment; ROC, receiver operating characteristics; Decision curve analysis.

**Figure 6:**
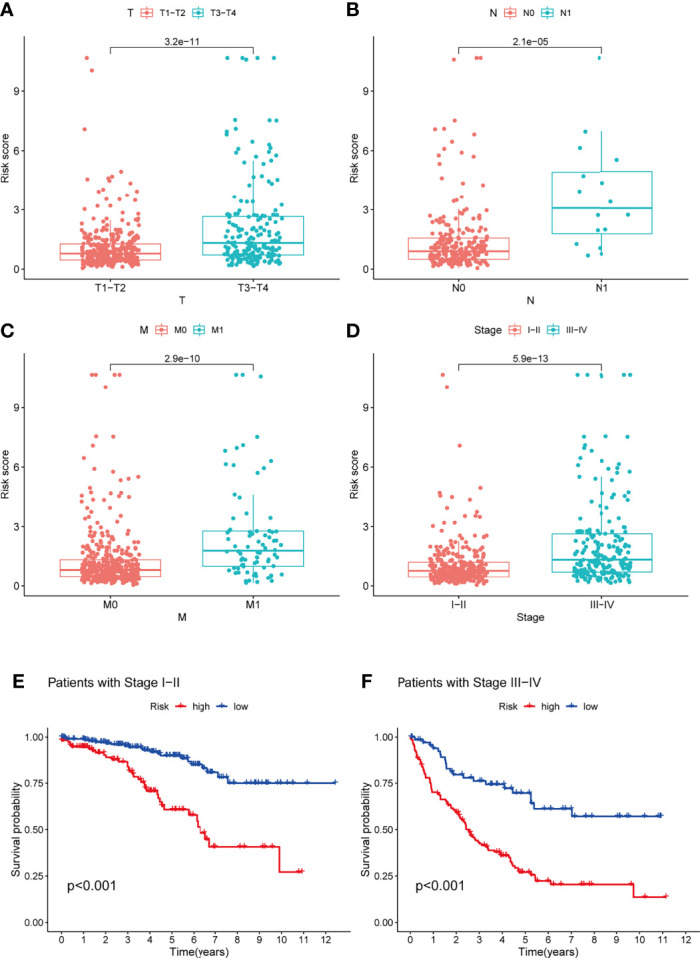
Analysis of the TME-related signature stratified by TNM staging and Stage staging. **(A–D)** Risk scores also tend to be higher in KIRC patients with combined T3-4, N1, M1, Stage III-IV (P < 0.05). **(E, F)** Patients with low-risk scores all had a better prognosis in the StageI-II and Stage III-IV groups (P < 0.05). TME, tumor microenvironment; KIRC, Kidney renal clear cell carcinoma.

The prognostic significance of different clinical characteristics was assessed using univariate and multivariate Cox regression analysis, and the results showed that risk score was an independent predictor of prognosis (**Table 3**). The ROC and DCA analysis found that the signature and nomogram were better predictors of patient prognosis than traditional clinical characteristics (**Figure 5**). Further stratification showed that risk scores increased with disease progression (**Figures 6A–D**) and were not affected by Stage (I-II and III-IV) (**Figures 6E, F**), and these results are sufficient to demonstrate the accuracy and stability of our signature.

**Table 3:** Univariable and multivariable Cox regression to analyze the relationship between the RS and clinical prognosis.

Variables	Univariable Analysis			Multivariable Analysis		
	HR	95%*CI*	*P-*Value	HR	95%*CI*	*P-*Value
Age	1.032	1.018–1.045	<0.001	1.031	1.017–1.046	<0.001
Gender	0.950	0.695–1.298	0.748	~	~	~
Grade	2.279	1.859–2.795	<0.001	1.311	1.042–1.651	0.021
Stage	1.863	1.633–2.126	<0.001	1.610	1.385–1.874	<0.001
Risk Score	1.273	1.220–1.328	<0.001	1.200	1.142–1.262	<0.001

### Comparison of Predictive Performance Between the TME-Related Signature and Previously Published Signatures

**Figure 7:**
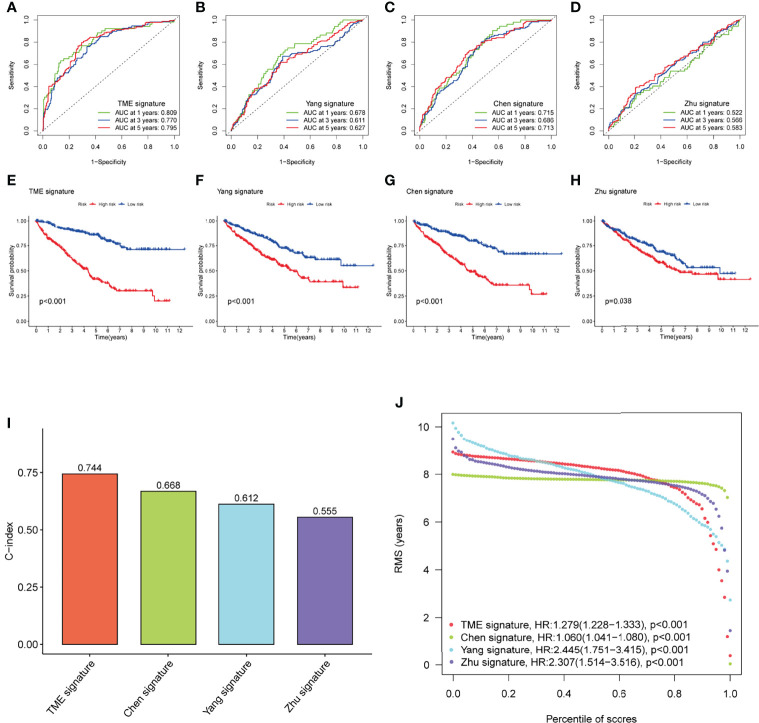
Performance of the TME-related signature compared with other prognostic signatures in predicting OS in KIRC patients. **(A–D)** ROC curves were used to predict 1-, 3-, and 5-year OS in KIRC patients with our signature and three other published gene signatures. **(E–H)** Kaplan-Meier survival curves of KIRC patients with our signature and three other published gene signatures. **(I)** The C-index of the four prognostic signatures including our signature, has the highest C-index. **(J)** The RMS time curves for our prognostic signature and the Chen/Zhu prognostic signatures showed an overlap of 8 years. TME, tumor microenvironment; OS, overall survival; KIRC, kidney renal clear cell carcinoma; C-index, concordance index; RMS, restricted mean survival.

To highlight the predictive performance of our 8-gene prognostic signature, three other established risk signatures were selected for comparison. The results showed that the AUC value of our signature was greater than those of the other three established prediction signatures we selected (**Figures 7A–D**) in predicting 1-, 3- and 5-year OS and that high-risk patients had a worse prognosis than low-risk patients (P<0.05, **Figures 7E–H**). The C-index of each model was calculated to evaluate the predictive power of the signature, and our signature had the highest C-index of 0.744 (**Figure 7I**). The restricted mean survival (RMS) results show that the model we have built performs best over a time of more than 8 years compared to the signatures built by Chen/Zhu (**Figure 7J**). The results demonstrate the robust predictive performance of our new signature.

### GSEA and Immune Correlation Analyses of the TME-Related Signature

**Figure 8:**
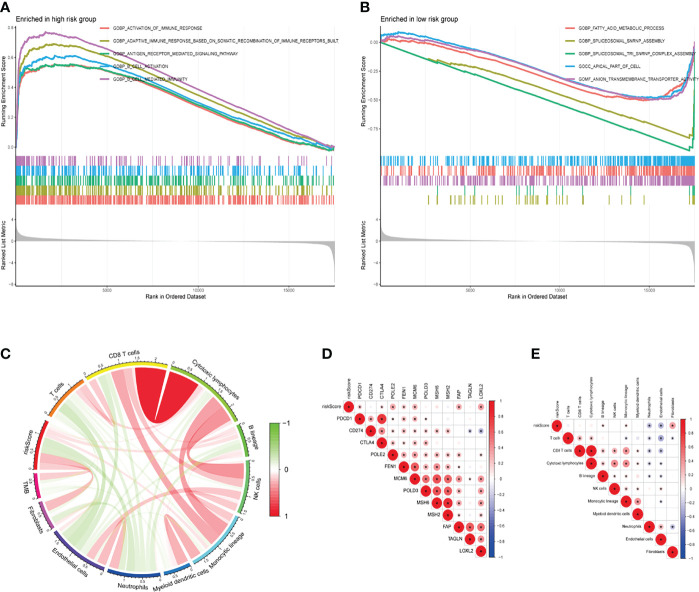
GSEA and immune correlation analyses of the TME-related signature. **(A, B)** GO-BP analyses for high- and low-risk groups. **(C)** Correlation between the risk score for our signature, immune cell score *via* the MCP-counter algorithm, and TMB (Pearson method). **(D)** The relationship between the risk score and expression of representative genes for immune checkpoints, DNA replication, mismatch repair, and epithelial-mesenchymal transition (Pearson method). **(E)** The relationship between the risk score and tumor immune infiltrating cells (Pearson method). GSEA, gene set enrichment analysis; TME, tumor microenvironment; GO, gene ontology; BP, biological process; MCP-counter, microenvironment cell populations-counter; TMB, tumor mutation burden.

GSEA analysis showed that high-risk group genes were mainly enriched in immune-related biological processes, such as activation of the immune response, adaptive immune response based on somatic ecombination of immune receptors built from immunoglobulin superfamily domains, antigen receptor mediated signalling pathway, B cell activation, B cell-mediated immunity (**Figure 8A**), the low-risk group genes were mainly enriched in fatty acid metabolic process, spliceosomal snrnp assembly, spliceosomal tri snrnp complex assembly, apical part of cell, anion transmembrane transporter activity (**Figure 8B**). Immunotherapy is now a treatment strategy with great potential after targeted therapy for patients with KIRC, and the tumor immune microenvironment is an important factor influencing the response to immunotherapy (20, 21). We used the MCP-counter algorithm to estimate immune cell scores for each patient with KIRC and then calculated their correlation with risk scores by Pearson’s method. The results showed that the risk score was significantly positively correlated with B lineage, Monocytic lineage, and Fibroblasts, while significantly negatively correlated with Neutrophils and Endothelial cells (**Figures 8C–E**). Interestingly, the risk score was also positively correlated with immune checkpoint-related genes (PDCD1, CTLA4), DNA replication-related genes (POLE2, FEN1, MCM6), and EMT-related genes (FAP, LOXL2) (**Figure 8D**).

## Discussion

Currently, the insidious onset of RCC and the lack of specific clinical signs in the early stages result in 20-30% of patients having metastases by the time of presentation (22). As KIRC has been studied more intensively, immunotherapy has been found to significantly improve the prognosis of patients (23–25). Results of completed clinical trials have shown various other immunomodulatory pathways in the tumor immune microenvironment, which can affect tumor cell survival and significantly influence immunotherapeutic response (26–28). Due to the heterogeneity of KIRC (29, 30) and immune relevance (31), the genetic information of TME is crucial to optimize the therapeutic approach of KIRC. In this study, we screened the differential genes based on the TME gene profile of KIRC And a prognostic index was constructed by association of these differential genes. This indicator is superior to those commonly used in the clinic and provides a prognostic method with lower sampling cost and better reproducibility for personalized treatment of patients.

In this study, we systematically analyzed 4061 TME-related genomic data from 537 KIRC cases in TCGA data to construct two clusters. The two clusters differed significantly in immune score comparisons for most tumor immune cells, as well as differences in survival prognostic analysis, which are consistent with the heterogeneity of the TME. The validated prognostic signature was constructed from eight TME-related genes (LRFN1, CSF1, UCN, TUBB2B, SERPINF1, ADAM8, ABCB4, CCL22) identified by univariate regression analysis and LASSO analysis. Patients were divided into a low-risk group and a high-risk group according to their risk scores. The difference in survival rates between the two groups was statistically significant, with the higher the risk score, the worse the prognosis of the patients. The stratified analysis revealed that the risk values increased with disease progression. The AUC values of our prognostic signature were almost >0.7, confirming its effectiveness in predicting the prognosis of KIRC patients. CSF1 acts as a tumour-promoting cytokine by recruiting macrophages to the tumour area, which in turn leads to the release of various tumour-promoting growth factors in the microenvironment (32). *In vitro* and *in vivo* studies have confirmed that CSF1 promotes proliferation and reduces apoptosis in renal cell carcinoma (33). TUBB2B alterations can lead to increased incidence of kidney disease and KIRC (34). Studies have shown that ADAM8 is highly expressed in a variety of malignant tumours and is strongly associated with tumour metastasis and poor patient prognosis (35, 36).

The nomogram is a practical and intuitive evaluation tool. The establishment of the nomogram can more accurately predict the prognosis of KIRC patients, and the ROC, DCA, and calibration curves showed the validity of the nomogram. Univariate and multifactorial Cox regression analyses combined with clinicopathological parameters showed this prognostic signature to be an independent prognostic factor for KIRC patients. When we compared our risk prognostic signature with the prognostic models developed by Yang et al (17), Chen et al (18), and Zhu et al (19), the overall performance of our signature was better than these three signatures.

Further biological process GSEA enrichment analysis was performed on patients in the high-risk and low-risk groups. The high-risk group was mainly enriched for several immune response-related biological processes. Interestingly, immune correlation analysis showed that risk values were positively correlated with B lineage, Monocytic lineage, Fibroblasts, and negatively correlated with Neutrophils, Endothelial cells. Monocytes are also involved in the immune response and will lose control of the tumour as it progresses, and the body’s defence system becomes less functional. Moreover, as a precursor to macrophages, high-risk patients have significantly higher levels of M0 macrophages relative to low-risk patients with KIRC (37). In recent years, immune checkpoints have received increasing attention in the study of tumour immunotherapy (38, 39). Kidney cancer is a highly immunogenic tumor in which the tumor cells produce an immunosuppressive environment through a variety of mechanisms, such as increased expression of immunosuppressive molecules(PDCD1, CD274, CTLA4) (40–42) in the TME and the occurrence of immune escape (43). Our analysis also confirmed that PDCD1 and CTLA4 expression levels were upregulated with increasing risk values, which may cause poor prognosis in patients at high risk of KIRC. In addition, our risk values were strongly and positively correlated with DNA replication-related genes (POLE2, FEN1, MCM6) and EMT-related genes (FAP, LOXL2). DNA replication-related genes play a crucial role in DNA replication and cell cycle regulation (44), and malfunctioning expression of these proteins has the potential to promote cell proliferation and tumorigenesis (45, 46). Previous studies have also shown that POLE2 and MCM6 are overexpressed in KIEC tissue and that high levels of POLE2 and MCM6 expression are associated with poor prognosis (47, 48). These immunosuppressive molecules and cell cycle regulatory genes may be involved in the development and progression of KIRC, informing its diagnosis and regression.

The Stage parameters need to be obtained from multiple imaging results and pathological staining and scored by a professional, our gene signature only requires the scoring of a specific gene after sequencing. This scoring process does not require the involvement of a professional and requires only simple calculations, thus providing better reproducibility and accessibility. And the gene information can be obtained from a small amount of tissue or even exosomes, thus greatly reducing the cost of obtaining these prognostic indicators. From the view in **Figure 5D**, the area under the AUC curve of our prognostic model is superior to all other clinically used parameters. The net benefit is also better than the stage indicator in the case of low risk. Therefore, our model is more advantageous and worthy of physicians’ choice. Patients in group C1 had higher immune scores for CD8+ T cells, cytotoxic lymphocytes, fibroblasts, monocyte lineage, myeloid dendritic cells, and T cells compared to group C2, while neutrophils and endothelial cells had lower immune scores instead, suggesting that the two subgroups C1 and C2 correspond to different TME immunophenotypes, respectively, and are potential indicators of immunophenotyping.

Admittedly, the current study still has some limitations that must be considered. This study is a retrospective study based on a public database and we need to validate these results in prospective cohorts, for animal models and at the cellular level, which will be the focus of our follow-up studies.

## Conclusion

In conclusion, this study successfully constructed a TME-related prognostic signature that accurately predicted the survival prognosis of KIRC patients. The nomogram established in combination with the risk score and other clinicopathological parameters can also individually predict patient survival. These results could provide the basis for future studies on potential individualized treatments for KIRC patients.

## Data Availability Statement

The origina contributions presented in the study are included in the article/**Supplementary Material**. Further inquiries can be directed to the corresponding author.

## Author Contributions

DP and CX contributed equally to this work. DP, CX and HZ designed this work. DW, XS and YL integrated and analyzed the data. DP, YZ, JG and NL wrote this manuscript. HZ and CX edited and revised the manuscript. All authors approved this manuscript.

## Funding

This study was funded by Henan Provincial Health Commission (LHGJ20190422), Henan Provincial Health Commission (LHGJ20210486) and the Key Scientific Item of Henan Province Education Department (21A320037).

## Conflict of Interest

The authors declare that the research was conducted in the absence of any commercial or financial relationships that could be construed as a potential conflict of interest.

The reviewer XJ declared a shared parent affiliation with the authors to the handling editor at the time of review.

## Publisher’s Note

All claims expressed in this article are solely those of the authors and do not necessarily represent those of their affiliated organizations, or those of the publisher, the editors and the reviewers. Any product that may be evaluated in this article, or claim that may be made by its manufacturer, is not guaranteed or endorsed by the publisher.

## Abbreviations

TME, Tumor microenvironment; KIRC, Renal clear cell carcinoma; NMF, non-negative matrix factorization; GSEA, Gene set enrichment analysis; RCC, Renal cell carcinoma; ECM, extracellular matrix; TAMs, tumour-associated macrophages; ICIs, immune checkpoint inhibition; TCGA, Cancer Genome Atlas; GEO, Gene-Expression Omnibus; OS, Overall survival; MCP-counter, microenvironment cell populations-counter; PFS, Progression-free survival; ROC, receiver operating characteristic; DCA, decision curve analysis; C-index, concordance index; BP, Biological process; EMT, epithelial-mesenchymal transition; GO, Gene Ontology; RMS, restricted mean survival; TMB, Tumor mutation burden
